# Supplementary material for: Systematic Review and Meta-Analysis of Metabolic Syndrome and Its Components in Latino Immigrants to the USA
Source: Int J Environ Res Public Health. 2023 Jan 11;20(2):1307. doi: 10.3390/ijerph20021307 (PMC9858988; doi:10.3390/ijerph20021307)
Supplement: Supplementary file 1 [file ijerph-20-01307-s001.zip › ijerph-2043373-supplementary/Supplemental files - updated after proofread/Table S3 - Search Strategy.pdf]

DATABASE<br>Website<br>Date of searches	STRATEGIES
PUBMED<br>https://pubmed.ncbi.nlm.nih.gov<br>June 1st, 2020	1. "Metabolic Syndrome"[Mesh] OR "metabolic syndrome"[TIAB] OR "Obesity"[Mesh] OR "obesity"[TIAB] OR "overweight"[MeSH Terms] OR "overweight"[TIAB] OR "waist circumference"[TW] OR "hypertension"[MeSH Terms] OR "hypertension"[TIAB] OR "dyslipidemias"[MeSH Terms] OR dyslipidemia*[TIAB] OR "hypertriglyceridemia"[MeSH Terms] OR "hypertriglyceridemia"[TIAB] OR "high triglycerides"[TIAB] OR "hyperglycaemia"[TIAB] OR "hyperglycemia"[MeSH Terms] OR "hyperglycemia"[TIAB] OR BMI OR “body mass index” OR “body weight” OR “Blood pressure” OR “high lipids” OR “HDL” OR "diabetes mellitus, type 2"[MeSH Terms] OR "type 2 diabetes mellitus"[TIAB] OR "diabetes type 2"[TIAB] OR "insulin resistance"[MeSH Terms] OR "insulin resistance"[TW] OR “sleep"[MeSH Terms] OR "sleep wake disorders"[MeSH] OR "sleep wake disorders"[TIAB] OR "sleep disorders"[TIAB]<br>2."Emigration and Immigration"[Mesh] OR Immigration[TW] OR Immigrant*[TIAB] OR Emigration[TIAB] OR migration[TW] OR migrant*[TIAB] OR “foreign-born”[TW] OR “country of origin”[TW] OR “region of birth”[TIAB] OR “region of origin”[TIAB]<br>3.America* [TIAB] OR USA[TIAB] OR "United States"[Mesh] OR “United States”[TIAB] OR AMERICAS[MESH] OR “North America”[TIAB]<br>4. Asia*[TIAB] OR European[TIAB] OR Indian[TIAB] OR Chinese[TIAB] OR Japanese[TIAB] OR Ukranian[TIAB] OR Korean[TIAB]<br>5. (#1 AND #2 AND #3) NOT #4<br>AND (("1980"[Date - Publication] : "3000"[Date - Publication]))
EMBASE<br>https://www.embase.com/<br>(Elsevier)<br>June 2nd 2020	1.'metabolic syndrome x'/exp OR 'obesity'/exp OR 'waist circumference'/exp OR 'hypertension'/exp OR 'dyslipidemia'/exp OR 'hypertriglyceridemia'/exp OR 'hyperglycemia'/exp OR 'non insulin dependent diabetes mellitus'/exp OR 'insulin resistance'/exp OR 'sleep'/exp OR 'sleep disorder'/exp<br>2. 'metabolic syndrome':ti,ab,kw OR 'obesity':ti,ab,kw OR 'overweight':ti,ab,kw OR 'waist circumference':ti,ab,kw OR 'hypertension':ti,ab,kw OR 'dyslipidemias':ti,ab,kw OR 'hypertriglyceridemia':ti,ab,kw OR 'high triglycerides':ti,ab,kw OR 'hyperglycaemia':ti,ab,kw OR 'hyperglycemia':ti,ab,kw OR 'bmi':ti,ab,kw OR 'body mass index':ti,ab,kw OR 'body weight':ti,ab,kw OR 'blood pressure':ti,ab,kw OR 'high lipids':ti,ab,kw OR 'hdl':ti,ab,kw OR 'type 2 diabetes mellitus':ti,ab,kw OR 'diabetes type 2':ti,ab,kw OR 'non insulin dependent diabetes mellitus':ti,ab,kw OR 'insulin resistance':ti,ab,kw OR 'sleep wake disorders':ti,ab,kw OR 'sleep disorders':ti,ab,kw<br>3. #1 OR #2<br>4. 'immigration'/exp OR 'migration'/exp<br>5. immigration:ti,ab,kw OR immigrant*:ti,ab,kw OR emigration:ti,ab,kw OR migration:ti,ab,kw OR migrant*:ti,ab,kw OR 'foreign-born':ti,ab,kw OR 'country of origin':ti,ab,kw OR 'region of birth':ti,ab,kw OR 'region of origin':ti,ab,kw<br>6. #4 OR #5<br>7. 'united states'/exp<br>8. america*:ab,ti OR usa:ab,ti OR 'united states':ab,ti OR 'north america':ab,ti<br>9. #7 OR #8<br>10. asia*:ti,ab,kw OR european:ti,ab,kw OR indian:ti,ab,kw OR chinese:ti,ab,kw OR japanese:ti,ab,kw OR ukranian:ti,ab,kw OR korean:ti,ab,kw<br>11. (#3 AND #6 AND #9) NOT #10

	#11 AND [embase]/lim NOT ([embase]/lim AND [medline]/lim) AND [1980-2020]/py
WEB OF SCIENCE https://clarivate.com/webofsciencegroup/solutions/web-of-science/ (Clarivate) June 2nd, 2020	1. TS=("Metabolic Syndrome" OR "obesity" OR "overweight" OR "waist circumference" OR "hypertension" OR dyslipidemia* OR "hypertriglyceridemia" OR "high triglycerides" OR "hyperglycaemia" OR "hyperglycemia" OR "BMI" OR "body mass index" OR "body weight" OR "Blood pressure" OR "high lipids" OR "HDL" OR "diabetes mellitus, type 2" OR "type 2 diabetes mellitus" OR "diabetes type 2" OR "non insulin dependent diabetes mellitus" OR "insulin resistance" OR "sleep wake disorders" OR "sleep disorders")<br>2.TS=(Immigration OR immigrant* OR emigration OR migration OR migrant* OR "foreign-born" OR "country of origin" OR "region of birth" OR "region of origin")<br>3.TS=(America* OR "USA" OR "United States" OR "North America")<br>4.TS=(Asia* OR European OR Indian OR Chinese OR Japanese OR Ukranian OR Korean)<br>5.(#1 AND #2 AND #3) NOT #4<br>Índices=SCI-EXPANDED, SSCI, A&HCI, CPCI-S, CPCI-SSH, ESCI Stipulated time=1980-2020
LILACS https://lilacs.bvsalud.org June 3rd, 2020	Strategy option 1:<br>("Metabolic Syndrome" OR "Sindrome metabolica" OR obesity OR obesidade OR overweight OR sobrepeso OR hypertension OR hypertension OR hipertensao OR dyslipidemia* OR triglycerides OR hyperglycemia OR triglicerid* OR hiperglicemia OR BMI OR "body mass index" OR "indice de massa corporal" OR "body weight" OR "Blood pressure" OR "pressão arterial" OR "presión arterial" OR HDL OR "diabetes mellitus" OR "sleep disorders") AND (Immigration OR immigrant* OR emigration OR migrant* OR imigrante* OR Inmigrante* OR inmigracao OR imigraçao OR emigraçao OR emigracion)<br><br>Strategy option 2:<br>(emigrantes or emigração or imigrante* or inmigrant or emigrant or migrant or migracao) AND ("metabolic syndrome" or "sindrome metabolica")
SciELO https://scielo.org June 3rd, 2020	(emigrantes or emigração or imigrante* or inmigrant or emigrant or migrant or migracao) AND ("metabolic
SCHOLAR GOOGLE https://scholar.google.com.br/ June 3rd, 2020	("metabolic syndrome" or obesity or diabetes or hypertension) and (migrant* or migration or immigrant* or immigration)
